# Effect of hemorrhage rate on early hemodynamic responses in conscious sheep

**DOI:** 10.14814/phy2.12739

**Published:** 2016-04-06

**Authors:** Christopher G. Scully, Chathuri Daluwatte, Nicole R. Marques, Muzna Khan, Michael Salter, Jordan Wolf, Christina Nelson, John Salsbury, Perenlei Enkhbaatar, Michael Kinsky, George C. Kramer, David G. Strauss

**Affiliations:** ^1^ Office of Science and Engineering LaboratoriesCenter for Devices and Radiological HealthUS Food and Drug AdministrationSilver SpringMaryland; ^2^ Department of AnesthesiologyThe University of Texas Medical BranchGalvestonTexas; ^3^ Office of Clinical Pharmacology, Office of Translational SciencesCenter for Drug Evaluation and ResearchUS Food and Drug AdministrationSilver SpringMaryland

**Keywords:** Hemodynamic compensation, hemorrhage, hypovolemia

## Abstract

Physiological compensatory mechanisms can mask the extent of hemorrhage in conscious mammals, which can be further complicated by individual tolerance and variations in hemorrhage onset and duration. We assessed the effect of hemorrhage rate on tolerance and early physiologic responses to hemorrhage in conscious sheep. Eight Merino ewes (37.4 ± 1.1 kg) were subjected to fast (1.25 mL/kg/min) and slow (0.25 mL/kg/min) hemorrhages separated by at least 3 days. Blood was withdrawn until a drop in mean arterial pressure (MAP) of >30 mmHg and returned at the end of the experiment. Continuous monitoring included MAP, central venous pressure, pulmonary artery pressure, pulse oximetry, and tissue oximetry. Cardiac output by thermodilution and arterial blood samples were also measured. The effects of fast versus slow hemorrhage rates were compared for total volume of blood removed and stoppage time (when MAP < 30 mmHg of baseline) and physiological responses during and after the hemorrhage. Estimated blood volume removed when MAP dropped 30 mmHg was 27.0 ± 4.2% (mean ± standard error) in the slow and 27.3 ± 3.2% in the fast hemorrhage (*P* = 0.47, paired *t* test between rates). Pressure and tissue oximetry responses were similar between hemorrhage rates. Heart rate increased at earlier levels of blood loss during the fast hemorrhage, but hemorrhage rate was not a significant factor for individual hemorrhage tolerance or hemodynamic responses. In 5/16 hemorrhages MAP stopping criteria was reached with <25% of blood volume removed. This study presents the physiological responses leading up to a significant drop in blood pressure in a large conscious animal model and how they are altered by the rate of hemorrhage.

## Introduction

Acute hemorrhage remains one of the leading causes of death for trauma patients with mortality frequently occurring within hours of initial injury (Dutton et al. 2010; Tisherman et al. 2015). This is of particular concern for teenagers and young adults for whom the leading causes of death are unintentional and intentional injuries (U.S. Centers for Disease Control and Prevention/National Center for Health Statistics, 2013). There remains a need to identify bleeding early and monitor a patient's physiology to, depending on the situation, ensure bleeding has stopped, prepare transfusions, or provide transfusions at the right time (Shackelford et al. 2014). This is a challenge due to complex and variable mammalian responses to hemorrhage.

The initial compensatory responses to reduced circulating blood volume due to hemorrhage is an increased heart rate and systemic vascular resistance in order to maintain perfusion to vital organs (Gutierrez et al. 2004). However, if blood loss continues, a maladaptive decompensatory hypotensive phase ensues (Gomez et al. 2012) that eventually results in insufficient oxygen delivery to organs and shock (Angele et al. 2008). It is imperative to identify hemorrhage early, as therapy is most effective in the compensatory phase. In general, a loss of 30% of blood volume is considered a tipping point from the sympathoexcitatory (compensatory) to sympathoinhibitory (decompensatory) phase of hemorrhage (Schadt and Ludbrook 1991). This corresponds with the Advanced Trauma Life Support (ATLS) classification from Class II (15–30%) to Class III (30–40%) hemorrhage. However, the exact volume of blood removed to shift from compensation to decompensation varies based on individual tolerances (Rickards et al. 2011) and variability in the injury (Kirkman and Watts 2014). Tolerance partially explains why the early stages of hemorrhage are often difficult to identify, since preservation of vital signs could be normal despite significant hemorrhage (1–1.5 L) (Bassin et al. 1971). Rickards et al. demonstrated that 1/3 of subjects undergoing lower body negative pressure experiments are “low” tolerant and enter the decompensatory phase at lower levels of central blood volume loss (Rickards et al. 2011). These individuals have been shown to have reduced baroreflex sensitivity and heart rates at the end of the compensatory phase, making it more difficult to identify when these subjects may reach a point of cardiovascular collapse (Schafer et al. 2013).

Injury variability from combined hemorrhage and tissue injuries can further complicate and alter the physiologic response to hemorrhage (Bassin et al. 1971; Kirkman and Watts 2014). A variable rate of hemorrhage results in a more severe response for the same fixed volume of blood removed in anesthetized animals (Frankel et al. 2007); however, anesthetized animals generally have an insufficient early compensatory phase to maintain blood pressure during low levels of blood volume loss (Frithiof et al. 2006). In conscious rats a faster hemorrhage alters the tachycardia response (Troy et al. 2003) and autonomic balance during the compensatory and decompensatory phases but does not affect the volume of blood removed to initiate decompensation (Porter et al. 2009). Increased tachycardia during a faster hemorrhage has also been shown to occur in conscious sheep (Starc and Stalcup 1986), and sheep may rely more on preservation of cardiac output rather than increased vascular resistance during the compensatory phase to maintain arterial pressure (Frithiof and Rundgren 2006). Therefore, we considered if altered compensatory responses due to varying hemorrhage rates translate to a meaningful change in the tolerance to hemorrhage in conscious sheep.

This study involved two hemorrhages at a slow (0.25 mL/kg_BW_/min or ~0.4% estimated blood volume [EBV] per minute) and fast (1.25 mL/kg_BW_/min or ~2% EBV per minute) hemorrhage rate in conscious sheep to test the hypothesis that the faster hemorrhage rate would result in reduced tolerance to hemorrhage and to assess differences in the early physiologic response to hemorrhage between these rates. Our model focuses on the response before a sudden decrease in MAP occurs, defined for our purposes as a drop in MAP of >30 mmHg from baseline. We also considered the differences in early cardiac and hemodynamic responses between hemorrhages where animals demonstrated low and high tolerance.

## Materials and Methods

### Animal preparation

A cross‐over study design was performed with conscious female Merino sheep (*N* = 8, 3 years old, weight: 33–40 kg) undergoing two hemorrhages separated by at least 3 days. Experiments were performed at the University of Texas Medical Branch under a protocol reviewed and approved by the Institutional Animal Care and Use Committee and conducted in compliance with the guidelines of the National Institutes of Health and the American Physiological Society for the care and use of laboratory animals. Sheep were purchased from Talley Ranch, Bastrop, TX and upon arrival at the research facility were held in quarantine for 15 days in 12‐h light/dark cycles and examined by a veterinarian for any possible preexisting pathologies. During this time sheep had access to food and water ad libitum. When ready for use, sheep were placed into metabolic cages and transferred to Translational Intensive Care Unit (TICU).

At the TICU, sheep were surgically prepared in a sterile operating room. Briefly, sheep were anesthetized with 5 mg/kg ketamine (KetaVed; Vedco Inc., St. Joseph, MO) followed by isoflurane (Piramal Healthcare Andhra Pradesh, India) inhalation (2–5%) via mask and endotracheal tube placement. Anesthesia was maintained with a mixture of isoflurane (2–5 vol%) in oxygen. Pre‐ and postsurgical analgesia was provided with long‐acting (72 h) buprenorphine (Buprenorphine SR; ZooPharm, Laramie, WY). Two arterial and venous lines were placed in left and right femoral vessels. A percutaneous introducer (9F Intro‐Flex; Centurion Medical Products Corp., Williamston, MI) was placed in the right jugular vein and used to advance a 7F Swan‐Ganz thermodilution catheter (131F7; Edwards Life Science, Irvine, CA) into the common pulmonary artery. Arterial lines and pulmonary arterial catheter were continuously flushed and connected to a transducer (Truwave PX4X4; Edwards Life Science, Irvine, CA) under pressure to prevent clotting. After surgical preparation, sheep were transferred to the intensive care unit and monitored for a core body temperature, complete blood cell count, discomfort, and pain. All vascular catheters were continuously flushed with heparinized saline throughout recovery and the study period to ensure patency. During surgical recovery, sheep were resuscitated with maintenance lactated Ringer's solution at a rate of 2 mL/kg_BW_/h.

### Experiments

After 7 days surgical recovery, each sheep underwent two hemorrhages on two different days separated by at least 3 days. On the day of the first hemorrhage the sheep was randomized to either a fast (1.25 mL/kg_BW_/min) or slow (0.25 mL/kg_BW_/min) hemorrhage rate. The selected hemorrhage rates were chosen to provide large differences in the expected timing of a sudden MAP drop and the terms fast (~2% EBV/min) and slow (~0.4% EBV/min) refer to their relationship with each other. The hemorrhage on the second day was performed at the alternate rate of the first day. Four animals were bled at the fast rate and four animals at the slow rate on their first experiment day. Water and maintenance fluids were removed 12 h before each experiment. All lines except the blood withdrawal line remained flushed with heparinized saline (~3 mL/h per line).

On the day of each experiment sheep were transferred to the operating room. They were able to sit and stand in the cage but could not turn around. The timeline of each study day is as follows: sensors placement period (~60 min), recovery period (60 min), baseline data collection (60 min), hemorrhage (experiment dependent), posthemorrhage (30 min), transfusion (experiment dependent), posttransfusion (30 min). For sensor placement, animals were sedated with an intravenous ketamine followed by isoflurane anesthesia via a mask. The following sensors were placed during this period (details on data recording instrumentation are below in “Instrumentation and measurements” section): urinary bladder catheter (Foley catheter with 4‐mm diameter oxygen tubing instead of conventional 8‐mm urine tubing to reduce dead space and risk of bladder retention), electrocardiogram (ECG) patches secured on shaved and naired front lower limbs and left rear limb, three tissue oximetry sensors (one on head and two on flank positions), respiratory belt, and pulse oximeter secured to tail by suture. During the sedation recovery period, a companion sheep in an isolated metabolic cage was brought into the operating room because sheep become anxious when left alone. Baseline data collection then occurred for 60 min as described in “Instrumentation and measurements” section.

Animals stood calmly in their cage during the experiments and did not show signs of pain or discomfort. During the study, animals would switch positions at times (lay down or stand up), and toward the end of the hemorrhage animals often lay down in their cage. The blood withdrawal rate was set using a large gauge sterile tubing rotary pump (MasterFlex Model 7518‐10; Cole‐Parmer, Vernon Hills, IL) and was initiated at the start of the hemorrhage period. The blood withdrawal was stopped when MAP dropped by 30 mmHg from baseline. Blood volume removed was estimated from the set flow rate on the pump and time until end of hemorrhage. Blood collection bags were also weighed for confirmation in 14 of 16 experiments. Data collection continued through a 30‐min posthemorrhage monitoring period. Withdrawn blood mixed with 250 mL normal saline was then reinfused at a rate of ~0.5 mL/kg_BW_/min until MAP reached baseline levels, followed by an additional 30‐min monitoring period. At the end of the first hemorrhage day for each animal, the remaining withdrawn blood was reinfused into the animal per gravity drop and hand pump. At the end of the second hemorrhage day, animals were euthanized under deep anesthesia and analgesia.

### Instrumentation and measurements

Continuous physiological waveforms were recorded using a 16‐channel data acquisition system in LabChart 7 Pro (ADInstruments Inc., Colorado Springs, CO) at a sampling rate of 1000 Hz. ECG, arterial pressure, pulmonary artery pressure, and central venous pressure were displayed in the operating room on a HP Hewlett Packard monitor M1176A Model 66. The position of the manometer was adjusted when the animal stood or lay down.

The tail pulse‐oximeter probe was connected to a Masimo Radical‐7 pulse oximeter (Masimo, Irvine, CA) and SpO_2_ was saved and downloaded from the pulse oximeter at 0.5 Hz. Tissue oximetry was measured from the forehead and two thigh locations (rSO_2‐CEREBRAL_, rSO_2‐THIGH1_, and rSO_2‐THIGH2_) with the Nonin SenSmart Model X‐100 (Nonin Medical, Inc., Plymouth, MN) and measurements were recorded at 0.25 Hz. The thigh locations were placed on the same side and in the same general location between animals. Oximeter data were stored on the devices and downloaded at the end of each experiment.

Intermittent measurements including thermodilution measurement of cardiac output and arterial and venous blood sampling were made at 60 min, 30 min, and 5 min before the start of hemorrhage, every 5 min (fast hemorrhage) or 20 min (slow hemorrhage) during the hemorrhage, 15 min and 30 min after the end of hemorrhage, and 15 min and 30 min after the end of the transfusion of blood. Pulmonary artery catheter thermodilution measurements of cardiac output were made using 10‐mL injections of iced saline. Two or three measurements were made at each time point and averaged for a single cardiac output value. Arterial blood sampling occurred from a femoral artery and mixed venous blood was sampled from the pulmonary artery catheter. Both were taken in 1 cc heparinized tuberculin syringe and analyzed in the operating room using a Siemens RAPIDPoint 500 (Siemens, Malvem, PA) for measurement of hematocrit, total hemoglobin, oxygen saturation (SO_2_), partial pressure of oxygen (PO_2_), partial pressure of carbon dioxide (PCO_2_), bicarbonate (HCO_3_), base excess, pH, and lactate. An additional 6–8 mL of blood was withdrawn and stored for additional analyses.

### Data analysis

All LabChart signals, CSV data files, and intermittent thermodilution and blood drawn measurements were analyzed in Matlab R2014b (The Mathworks, Natick, MA). Waveforms recorded at 1000 Hz were low‐pass filtered with an antialiasing filter and downsampled to 250 Hz for analysis. Blood pressure pulses were identified using a custom pulse detector to derive heart rate, systolic, diastolic, mean, and pulse pressures. Reported vital sign measurements are 1 min means unless otherwise noted.

Stroke volume was determined as (cardiac output/heart rate) and total peripheral resistance as (MAP/cardiac output). Body surface area (BSA) was estimated using standard formulas, and cardiac index is reported as (cardiac output/BSA) and stroke volume index as (stroke volume/BSA). Initial blood volume was estimated at 60 mL/kg_BW_. To study the differences in physiological responses leading to a 30 mmHg MAP decrease, data during the hemorrhage period were aligned as the percent estimated blood volume removed (EBVR) divided by the maximum estimated blood volume removed (EBVR_MAX_ – blood volume at the end of hemorrhage resulting in a 30 mmHg MAP decrease) for each experiment. This normalized estimate is referred to as EBVR/EBVR_MAX_ and ranges from 0% (at the start of the hemorrhage) to 100% (at the end of hemorrhage).

### Statistics

Unless otherwise noted, data are displayed as mean ± standard error (SE). The Shapiro–Wilk test was used to verify normal distributions of all measured variables. Effect of day of test on each physiological parameter was studied using paired *t* test. During hemorrhage, effects of hemorrhage rate, EBVR/EBVR_MAX_, on each physiological parameter relative to its baseline measurement was examined using linear growth curve model with a compound symmetry variance–covariance structure to incorporate correlations for all of the observations arising from the same animal using the PROC MIXED procedure in SAS (Version 9.4, SAS Institute Inc., Cary, NC). Denominator degrees of freedom for *F* tests for fixed effects were estimated using inflated variance matrix suggested by Kenward and Roger DDFM = KR in PROC MIXED procedure (Brown and Prescott 2006). The effect of hemorrhage rate at each EBVR/EBVR_MAX_ level was examined using tests of simple effects (Winer et al. 1971) LSMEANS statement with option SLICE in SAS. During recovery after hemorrhage, effects of hemorrhage rate, and time since end of hemorrhage, on each physiological parameter were examined using similar linear growth curve model with a compound symmetry variance–covariance structure to incorporate correlations for all of the observations arising from the same animal using the PROC MIXED procedure in SAS. The effect of hemorrhage rate at each time point since the end of hemorrhage was examined using tests of simple effects (Winer et al. 1971) LSMEANS statement with option SLICE in SAS.

Each intermittent measurement point (from blood draws and thermodilution) is converted to normalized estimated blood volume removed (EBVR) as current estimated blood removed divided by the estimated blood volume removed at the end of hemorrhage (EBVR/EBVR_MAX_). Intermittent measurements during the hemorrhage is then grouped by EBVR/EBVR_MAX_ as (baseline, >0–50%, >50–90%, >90–100%) to provide an even distribution of data points from each experiment representative of the different levels of blood loss. Outside of the hemorrhage period, data are grouped by time of measurement (baseline, end of hemorrhage [EOH], EOH + 15 min, and EOH + 30 min).

## Results

Fast and slow hemorrhages were performed on the same animals so that the weight (37.4 ± 1.1 kg) and estimated initial blood volume (2243 ± 61 mL) were the same for both rates. Baseline measurements averaged over the last 20 min before the start of hemorrhage for hemodynamic parameters are reported in Table 1. No significant differences were observed in baseline measurements between rates. Due to sensor malfunctions data were not available from all sensors for all hemorrhage trials. The number of experiments being reported for each measurement is reported in Table 1.

**Table 1:** Baseline measurements

	Slow (0.25 mL/kg_BW_/min)	Fast (1.25 mL/kg_BW_/min)
Mean arterial pressure (mmHg)	99.7 ± 3.9 (8)	96.3 ± 3.2 (8)
	[84.4, 112.3]	[81.7, 110.6]
Systolic arterial pressure (mmHg)	120.7 ± 3.4 (8)	120.4 ± 4.1 (8)
	[106.7, 139.2]	[100.7, 133.9]
Diastolic arterial pressure (mmHg)	89.1 ± 4.6 (8)	84.2 ± 3.3 (8)
	[72.3, 106.3]	[72.2, 99.0]
Heart rate (bpm)	102.2 ± 5.9 (8)	105.9 ± 4.9 (8)
	[85.7, 133.4]	[91.1, 127.1]
Pulse pressure (mmHg)	31.6 ± 3.6 (8)	36.2 ± 3.0 (8)
	[11.2, 44.6]	[25.3, 50.9]
Central venous pressure (mmHg)	7.7 ± 1.9 (8)	7.5 ± 1.1 (7)
	[2.1, 15.7]	[4.2, 12.9]
Mean pulmonary artery pressure (mmHg)	16.7 ± 2.4 (8)	21.8 ± 1.5 (7)
	[6.1, 28.9]	[17.5, 27.2]
Cardiac index (L/min/m^2^)	7.41 ± 0.28 (8)	7.28 ± 0.53 (7)
	[6.3, 8.4]	[6.1, 10.1]
Stroke volume index (mL/m^2^)	73.1 ± 3.6 (8)	71.7 ± 6.4 (7)
	[50.6, 82.8]	[59.7, 107.5]
Total peripheral resistance (dyn·sec·cm^−5^ m^−2^)	1162 ± 71 (8)	1155 ± 81 (7)
	[896, 1539]	[820, 1474]
SpO_2_ (%)	95.8 ± 1.3 (7)	97.0 ± 1.0 (6)
	[90.9, 99.8]	[92.7, 99.6]
rSO_2‐CEREBRAL_ (%)	61.1 ± 3.6 (6)	67.1 ± 4.4 (7)
	[49.0, 70.4]	[48.5, 84.4]
rSO_2‐THIGH1_ (%)	72.3 ± 3.6 (3)	64.9 ± 2.1 (6)
	[65.5, 77.5]	[55.2, 69.3]
rSO_2‐THIGH2_ (%)	68.6 ± 2.2 (3)	73.5 ± 4.1 (4)
	[64.9, 72.5]	[63.8, 82.1]

Values are mean ± SE (*N*), [min, max].

### Effect of hemorrhage rate on the tolerance to blood loss

**Figure 1:**
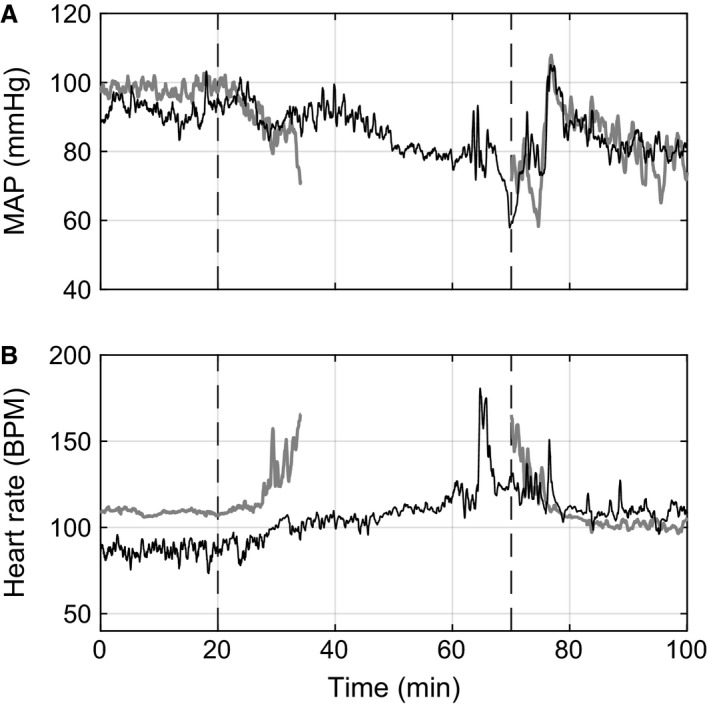
(A) Mean arterial pressure (MAP) and (B) heart rate during the slow (thin black line) and fast (thick gray line) hemorrhages for one animal. First dashed vertical line indicates the start of hemorrhage and second vertical line indicates the end of the longer hemorrhage and beginning of the posthemorrhage period.

Mean arterial pressure (MAP) and heart rate responses from one animal during and after the hemorrhage are presented in Figure 1. Due to the varying hemorrhage rates the slow hemorrhage occurs over a longer time.

**Figure 2:**
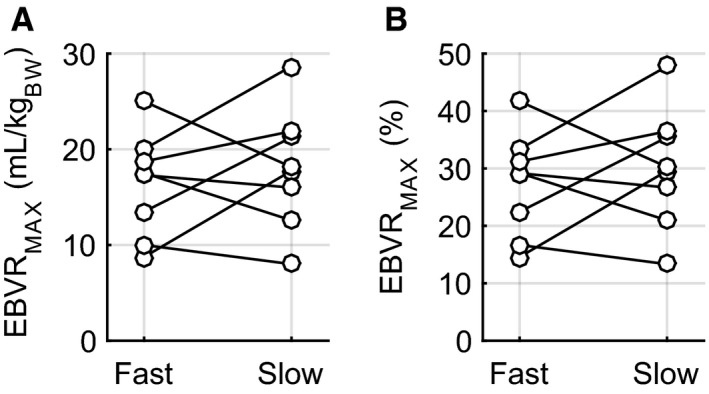
Individual volumes of blood removed for each animal during fast and slow hemorrhage rates: (A) mL/kg_BODY__‐_ _WEIGHT_ (B) percent (%) of total estimated blood volume.

The decrease in MAP measured at the time hemorrhage was stopped was not affected by the rate of hemorrhage (Slow: 33.4 ± 1.0 mmHg vs. Fast: 32.4 ± 2.5 mmHg, *P* = 0.37). Slow hemorrhage experiments took significantly longer time to reach the hemorrhage stopping point (Slow: 72.1 ± 8.8 min vs. Fast: 13.3 ± 1.5 min, *P* < 0.001), but the volume (Slow: 18.0 ± 2.2 mL/kg_BW_ vs. Fast: 16.4 ± 1.9 mL/kg_BW_, *P* = 0.25) and percentage (Slow: 30.1 ± 3.7% vs. Fast: 27.3 ± 3.2%, *P* = 0.25) of total blood removed (EBVR_MAX_) were equivalent between rates (Fig. 2). The actual volume of blood required to reach the stopping point varied considerably among experiments for both rates (Fig. 2). The minimum and maximum percentage of blood removed to reach the stopping point were 13.3% and 47.8%, respectively, for the slow hemorrhage and 14.6% and 41.7% for the fast hemorrhage. Due to the slow/fast randomization between hemorrhage days, there was no significant difference between experiment days in the time to reach the end of hemorrhage (Day 1: 45.8 ± 12.2 min vs. Day 2: 39.6 ± 13.3 min, *P* = 0.25), total blood volume removed (Day 1: 18.7 ± 1.6 mL/kg_BW_ vs. Day 2: 15.7 ± 2.3 mL/kg_BW_, *P* = 0.16), or percent blood volume removed (Day 1: 31.1 ± 2.7% vs. Day 2: 26.2 ± 3.9%, *P* = 0.16).

### Effect of hemorrhage rate on hemodynamic and oxygenation response

**Figure 3:**
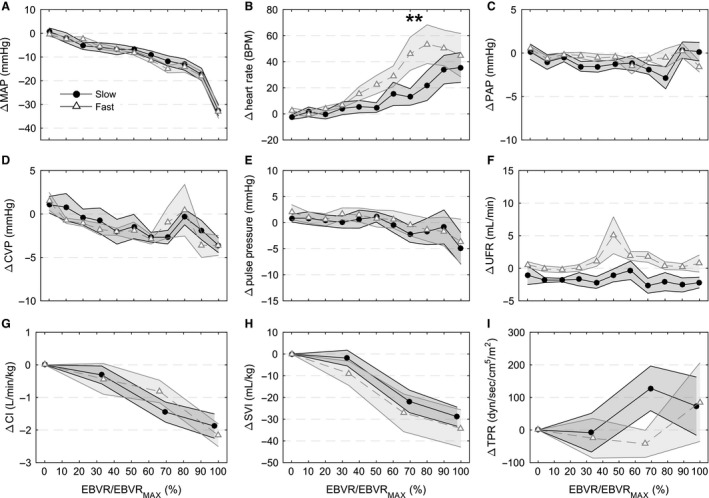
Hemodynamic changes during hemorrhage with respect to baseline measurements. (A) mean arterial pressure (MAP), (B) heart rate, (C) mean pulmonary artery pressure (PAP), (D) central venous pressure (CVP), (E) pulse pressure, (F) urine flow rate (UFR), (G) cardiac index (CI), (H) stroke volume index (SVI), and (I) total peripheral resistance (TPR). Horizontal axis represents the estimated blood volume removed as a percentage of the total estimated blood volume removed when MAP decreased 30 mmHg and the hemorrhage ended (EBVR/EBVR_MAX_). 0% is the start of hemorrhage and 100% the end of hemorrhage. Open gray triangles (fast hemorrhage rate) and solid circles (slow hemorrhage rate) represent the mean and shaded regions standard error (for CVP, CI, SVI, and TPR fast *N* = 7, all other groups *N* = 8). * indicates significant difference (*P* < 0.05) between fast and slow hemorrhage rates at specified level of EBVR/EBVR_MAX_.

Mean arterial pressure responses during hemorrhage were similar between the two rates. There was a gradual decrease in MAP throughout most of the hemorrhage followed by a sudden drop over the final 10% of blood removal (Fig. 3A). During the slow hemorrhage, MAP decreased 18.1 ± 2.0 mmHg through 90% of blood volume removal compared to 15.3 ± 2.4 mmHg during the final 10% of blood volume removal (45.3 ± 0.1% of the total MAP decrease occurred during the final 10% of blood volume removed), signifying an end of the initial compensatory response to hemorrhage. This pattern was the same during the fast hemorrhage: 15.0 ± 3.8 mmHg of the 32.4 ± 2.5 mmHg (44.2 ± 0.1%) MAP decrease during hemorrhage occurred during the final 10% of blood volume removed.

Tachycardia was present during hemorrhages at both rates. The peak heart rate reached during the hemorrhage was not different between the hemorrhage rates (Slow: 156 ± 11 bpm vs. Fast: 172 ± 19 bpm, *P* = 0.17) (computed from 20 sec averaged data to better identify the peak value). Heart rates were higher during the fast hemorrhage compared to the slow hemorrhage at earlier levels of EBVR/EBVR_MAX_ (Fig. 3B). Central venous pressure (CVP) decreased during both hemorrhages (Fig. 3D). Note that the results plotted in Figure 3 are with respect to the baseline measurements averaged over the 10 min preceding the start of hemorrhage, while the point at the EBVR/EBVR_MAX_ of 0% is taken at the start of hemorrhage. This results in the *y*‐axis values not being at 0 for an EBVR/EBVR_MAX_ of 0%.

Intermittent measurements (thermodilution and blood draws) during the hemorrhage were grouped into three clusters to account for the varying hemorrhage rates and hemorrhage tolerance: 0–50%, 50–90%, and 90–100%. EBVR/EBVR_MAX_ was equivalent between the two hemorrhage rates in the 0–50% (Slow: 32.7 ± 3.2% vs. Fast: 34.4 ± 3.0%), 50–90% (Slow: 70.6 ± 3.5 vs. Fast: 66.5 ± 2.3%), and 90–100% (Slow: 97.6 ± 0.8% vs. Fast: 95.69 ± 0.8%) measurement zones.

Thermodilution measurements of cardiac output were not available for one fast hemorrhage. The early increase in heart rate helped to stabilize cardiac output while stroke volume decreased (Fig. 3G). Total peripheral resistance did not change significantly for either rate during the hemorrhage (Fig. 3I); however, there were substantial differences in the response of total peripheral resistance based on individual tolerance that is presented in “Response of low‐tolerant experiments” section. There was no difference in hemoglobin between hemorrhage rates at baseline (Slow: 7.36 ± 0.53 g/dL vs. Fast: 8.04 ± 0.40 g/dL, *P* = 0.25), but it was stable during the slow and increased during the fast hemorrhage (Table 2). The largest differences with respect to hemorrhage rate in hemoglobin occurred at 50–90% EBVR/EBVR_MAX_ (Slow: 7.84 ± 0.41 vs. Fast: 9.08 ± 0.41 g/dL, *P* < 0.01) and 90–100% EBVR/EBVR_MAX_ (Slow: 7.16 ± 0.46 vs. Fast: 8.59 ± 0.45 g/dL, *P *≤ 0.04).

**Table 2:** Intermittent blood drawn measurements

	Rate	Baseline	EBVR/EBVR_MAX_			Time since end of hemorrhage		
			>0–50%	50–90%	90–100%	0 min	15 min	30 min
Hemoglobin^b^ (g/dL)	Slow	7.36 ± 0.53 (8)	7.40 ± 0.54 (11)	7.84 ± 0.41 (12)	7.16 ± 0.46 (10)	7.24 ± 0.50 (7)	6.66 ± 0.36 (8)	6.33 ± 0.39 (7)
	Fast	8.04 ± 0.40(7)	8.38 ± 0.66 (6)^c^	9.08 ± 0.41 (8)^c^	8.59 ± 0.45 (7)^c^	8.59 ± 0.45 (7)^c^	7.64 ± 0.36 (7)	7.2 ± 0.40 (7)
Hematocrit^b^ (%)	Slow	21.8 ± 1.8 (8)	21.7 ± 1.8 (11)	23.2 ± 1.3 (12)	21.1 ± 1.5 (10)	21.3 ± 1.7 (7)	19.4 ± 1.2 (8)	18.5 ± 1.4 (7)
	Fast	23.5 ± 1.2 (7)	24.5 ± 2.0 (6)^c^	26.9 ± 1.2 (8)^c^	25.1 ± 1.3 (7)^c^	25.1 ± 1.3 (7)^c^	22.4 ± 1.0 (7)^c^	21.3 ± 1.1 (7)
SaO_2_ (%)	Slow	96.0 ± 1.2 (8)	97.6 ± 0.5 (11)	97.6 ± 0.6 (12)	96.8 ± 0.8 (10)	97.0 ± 0.6 (7)	96.9 ± 1.2 (8)	98.0 ± 0.5 (7)
	Fast	96.0 ± 1.0 (7)	96.8 ± 0.4 (6)	96.0 ± 1.5 (8)	93.1 ± 4.0 (7)	93.1 ± 4.0 (7)	96.3 ± 1.2 (7)	95.2 ± 1.3 (7)
SvO_2_ a,b (%)	Slow	60.5 ± 2.8 (8)	60.7 ± 3.7 (11)	64.0 ± 5.4(12)	44.0 ± 3.5 (11)	44.4 ± 4.5 (8)	47.2 ± 4.5(8)	51.9 ± 5.3 (7)
	Fast	58.0 ± 2.3 (8)	56.4 ± 4.3 (7)	51.0 ± 3.6 (8)^c^	40.5 ± 5.4 (8)	40.5 ± 5.4 (8)	41.4 ± 3.7 (8)^c^	48.5 ± 3.8 (8)
DO_2_ a,b (mL O_2_/min)	Slow	623 ± 37 (7)	643 ± 76 (11)	572 ± 40 (12)	470 ± 34 (10)	473 ± 38 (7)	404 ± 30 (8)	471 ± 50 (7)
	Fast	702 ± 91 (6)	745 ± 87 (6)	745 ± 71 (6)^c^	510 ± 63 (7)	510 ± 63 (7)	430 ± 53 (7)	473 ± 55 (7)
VO_2_ (mL O_2_/min)	Slow	225 ± 26 (7)	244 ± 36 (11)	196 ± 34 (12)	257 ± 24 (10)	257 ± 28 (7)	203 ± 19 (8)	227 ± 43 (7)
	Fast	290 ± 21 (6)	276 ± 13 (6)	327 ± 27 (6)^c^	278 ± 25 (7)	278 ± 25 (7)	245 ± 25 (7)	228 ± 21 (7)
O_2_ER^a^,^b^ (%)	Slow	37.2 ± 2.8 (7)	37.8 ± 3.8 (11)	34.4 ± 5.5 (12)	54.7 ± 4.0 (10)	54.5 ± 5.3 (7)	51.1 ± 4.8 (8)	46.9 ± 5.6 (7)
	Fast	40.7 ± 2.2 (6)	39.4 ± 4.4 (6)	47.8 ± 4.0 (6)^c^	57.8 ± 5.9 (7)	57.8 ± 5.9 (7)	58.8 ± 3.9 (7)	50.3 ± 4.4 (7)
PO_2_ (mmHg)	Slow	92.0 ± 7.6 (8)	95.1 ± 4.2 (11)	96.2 ± 4.0 (12)	88.4 ± 5.0 (10)	89.8 ± 5.1 (7)	98.6 ± 4.2 (8)	100.4 ± 8.2 (7)
	Fast	85.5 ± 4.4 (7)	84.7 ± 4.0 (6)	89.8 ± 7.2 (8)	84.7 ± 8.9 (7)	84.7 ± 8.9 (7)	86.7 ± 5.4 (7)^c^	88.2 ± 5.2 (7)
PCO_2_ (mmHg)	Slow	30.8 ± 1.2 (8)	32.2 ± 1.2 (11)	31.1 ± 0.8 (12)	33 ± 0.8 (10)	32.4 ± 0.9 (7)	31.8 ± 1.0 (8)	31.3 ± 1.8 (7)
	Fast	32.2 ± 0.6 (7)	32.4 ± 0.7 (6)	31.3 ± 1.1 (8)	30.5 ± 1.5 (7)	30.5 ± 1.5 (7)	30.5 ± 1.0 (7)	30.7 ± 1.0 (7)
Lactate^b^ (mmol/L)	Slow	0.70 ± 0.18 (8)	0.64 ± 0.12 (11)	0.59 ± 0.09 (12)	0.58 ± 0.10 (10)	0.63 ± 0.14 (7)	0.65 ± 0.09 (8)	0.59 ± 0.08 (7)
	Fast	0.76 ± 0.13 (7)	0.59 ± 0.08 (6)	0.74 ± 0.10 (8)^c^	0.80 ± 0.12 (7)	0.80 ± 0.12 (7)	1.22 ± 0.22 (7)^c^	0.88 ± 0.16 (7)^c^
HCO_3_ (mmol/L)	Slow	24.1 ± 0.6 (8)	25.4 ± 0.8 (11)	24.5 ± 0.4 (12)	25.1 ± 0.4 (10)	24.7 ± 0.4 (7)	24.5 ± 0.3 (8)	24.4 ± 0.9 (7)
	Fast	25.0 ± 0.9 (7)	25.6 ± 1.0 (6)	24.8 ± 0.9 (8)	23.6 ± 0.8 (7)	23.6 ± 0.8 (7)	23.9 ± 0.9 (7)	24.1 ± 0.9 (7)
Base excess (mmol/L)	Slow	1.3 ± 0.5 (8)	2.5 ± 0.7 (11)	1.7 ± 0.4 (12)	2.1 ± 0.4 (10)	1.6 ± 0.4 (7)	1.4 ± 0.3 (8)	1.4 ± 0.8 (7)
	Fast	2.0 ± 1.0 (7)	2.6 ± 1.1 (6)	2.1 ± 0.9 (8)	0.8 ± 0.8 (7)	0.8 ± 0.8 (7)	1.0 ± 1.0 (7)	1.2 ± 0.9 (7)
pH	Slow	7.51 ± 0.01 (8)	7.51 ± 0.01 (11)	7.51 ± 0.01 (12)	7.51 ± 0.01 (10)	7.50 ± 0.01 (7)	7.51 ± 0.01 (8)	7.51 ± 0.02 (7)
	Fast	7.51 ± 0.02 (7)	7.51 ± 0.01 (6)	7.52 ± 0.01 (8)	7.51 ± 0.02 (7)	7.51 ± 0.02 (7)	7.51 ± 0.01 (7)	7.51 ± 0.01 (7)

Values are means ± SE (number of measurements within each interval).

a Significant effect of percent blood withdrawn (*P* < 0.05).

b significant effect of time since end of hemorrhage (*P* < 0.05).

c significant effect of hemorrhage rate at specified percent blood withdrawn or time since end of hemorrhage (*P* < 0.05).

**Figure 4:**
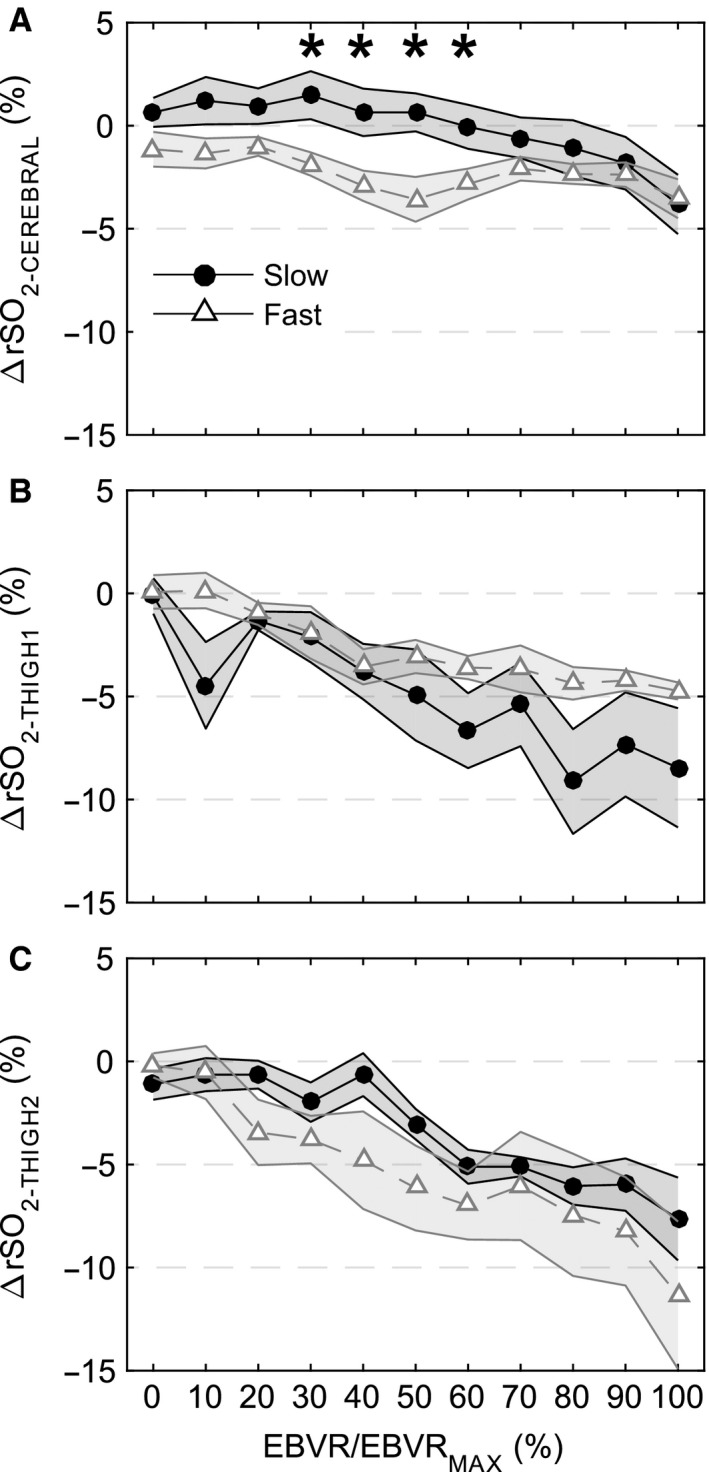
Regional oximetry measurements with respect to baseline during hemorrhage plotted against EBVR/EBVR_MAX_. (A) rSO2_‐_ _CEREBRAL_ (Slow *N* = 6, Fast *N* = 7), (B), rSO2_‐_ _THIGH__1_ (Slow *N* = 3, Fast *N* = 6), and (C) rSO2_‐_ _THIGH__2_ (Slow *N* = 3, Fast *N* = 4). Open gray triangles (fast hemorrhage rate) and solid black circles (slow hemorrhage rate) represent the mean and shaded regions standard error of the mean. * indicates significant difference between fast and slow hemorrhage rate at specified level of EBVR/EBVR_MAX_.

Arterial oxygen saturation and breathing rate did not change during the hemorrhage for either rate (data not shown). A progressive decrease in tissue oxygenation was observed from rSO_2‐THIGH1_, and rSO_2‐THIGH2_ measurements (Fig. 4B andC), however, due to technical problems with the sensors these were only recorded during a limited number of experiments. rSO_2‐CEREBRAL_ was stable until the end of the slow hemorrhage, but showed a slight early decrease followed by recovery during the fast hemorrhage (Fig. 4A). The decrease in tissue oximetry corresponded to a decrease in venous oxygen saturation (SvO_2_) and increase in the oxygen extraction ratio (O_2_ER) toward the end of the hemorrhage (Table 2). At 50–90% EBVR/EBVR_MAX_ SvO_2_ was significantly lower in the fast hemorrhage (Slow: 64.0 ± 5.4 vs. Fast: 51.0 ± 3.6, *P* = 0.014) and O_2_ER was significantly higher (Slow: 34.4 ± 5.5 vs. Fast: 47.8 ± 4.0, *P* = 0.024). Changes in SvO_2_ and O_2_ER did not occur until the end of blood withdrawal during the slow hemorrhage.

### Posthemorrhage recovery

**Figure 5:**
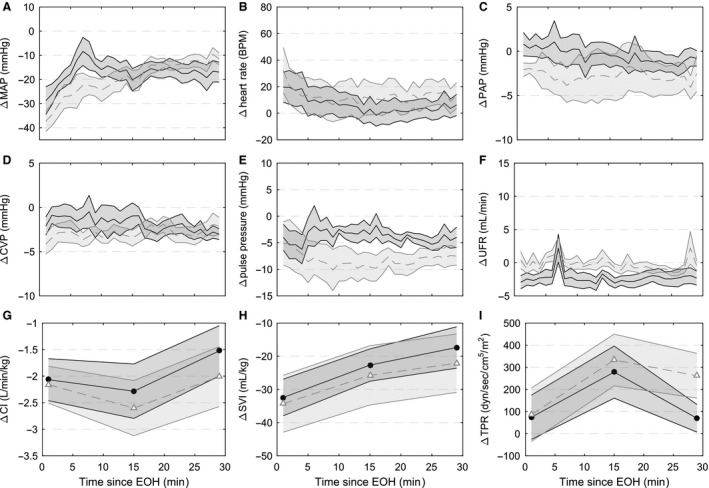
Hemodynamics during the 30 min after the end of hemorrhage (EOH) displayed as the difference from baseline measurements. (A) Mean arterial pressure (MAP), (B) heart rate, (C) mean pulmonary artery pressure (PAP), (D) central venous pressure (CVP), (E) pulse pressure, (F) urine flow rate (UFR), (G) cardiac index (CI), (H) stroke volume index (SVI), and (I) total peripheral resistance (TPR). Dashed gray (fast hemorrhage rate) and solid black (slow hemorrhage rate) center lines and shaded regions represent the mean and standard error of the mean.

The heart rate and hemodynamic responses for 30 min after the hemorrhage ended are presented in Figure 5. During the posthemorrhage period, MAP recovered more quickly after the slow hemorrhage than the fast hemorrhage, but by 15 min posthemorrhage there was no effect of rate on the MAP difference with respect to baseline (Slow: 19.6 ± 4.6 mmHg vs. Fast: 20.0 ± 4.2 mmHg at EOH + 15 min time point). By 30‐min posthemorrhage, the MAP difference with respect to baseline recovered to 16.2 ± 4.1 mmHg for the slow hemorrhage and 10.9 ± 2.4 mmHg for the fast hemorrhage. Heart rate dropped from its peak posthemorrhage for both fast and slow rates. Pulse pressure continued to decrease after the fast hemorrhage resulting in a significantly lower pulse pressure compared to the slow hemorrhage (*P* < 0.001) for most of the posthemorrhage period. CVP was also lower after the fast compared to the slow hemorrhage for most of the posthemorrhage period. Cardiac index and stroke volume index showed modest recoveries after both hemorrhage rates (Fig. 5G and H). This at least partly resulted from an increase in TPR that occurred during the posthemorrhage period, countering the decrease in heart rate (Fig. 5I). Decreased hemoglobin and hematocrit posthemorrhage for both rates suggest a redistribution of blood from tissues to vessels.

**Figure 6:**
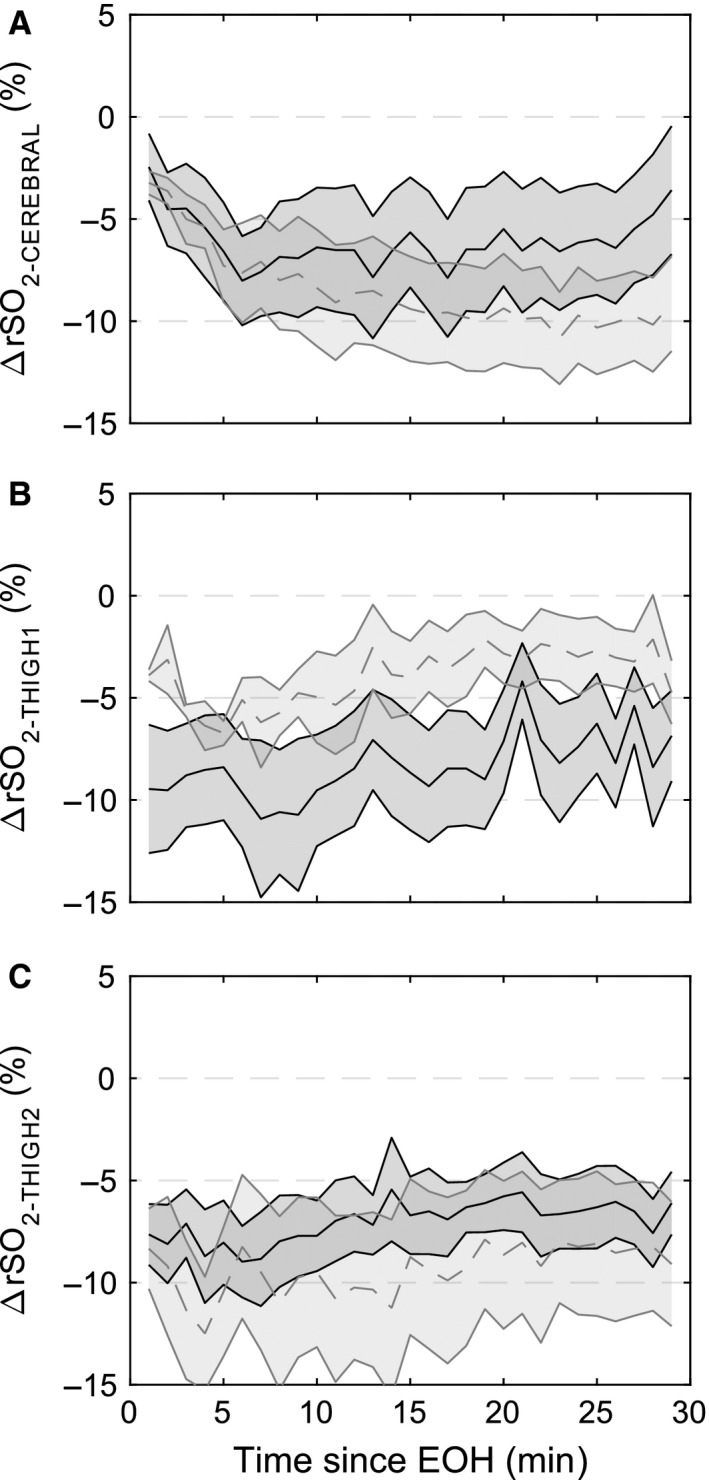
Regional oximetry measurements with respect to baseline during the recovery period against time since the end of hemorrhage (EOH). (A) rSO2_‐_ _CEREBRAL_ (Slow *N* = 6, Fast *N* = 7), (B), rSO2_‐_ _THIGH__1_ (Slow *N* = 3, Fast *N* = 6), and (C) rSO2_‐_ _THIGH__2_ (Slow *N* = 3, Fast *N* = 4). Gray dashed (fast hemorrhage rate) and black solid (slow hemorrhage rate) center lines and shaded regions represent the mean and standard error of the mean.

SaO_2_ did not change posthemorrhage, while SvO_2_ and O_2_ER recovered some over the 30‐min monitoring period (Table 2). This corresponded to a gradual increase in rSO_2‐THIGH1_ and rSO_2‐THIGH2_, while rSO_2‐CEREBREAL_ continued to decrease after the hemorrhage ended (Fig. 6). Lactate measurements were elevated only in the fast hemorrhage group at the 15‐ and 30‐min measurement points posthemorrhage, while pH was stable throughout the experiments for both hemorrhage rates.

### Response of low‐tolerant experiments

**Figure 7:**
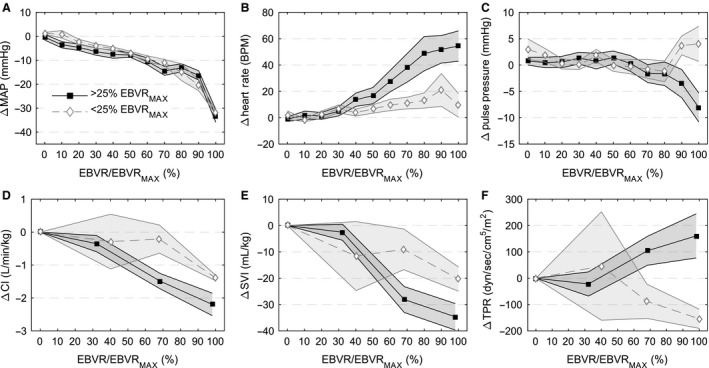
Hemodynamic responses during low‐ (open diamonds, *N* = 5) and high‐ (solid squares, *N* = 11) tolerant hemorrhages. Low‐tolerant hemorrhages are defined here as those with a 30 mmHg MAP decrease from baseline occurring with <25% blood volume removed. Solid center lines are mean and shaded areas are standard error. Data are presented as difference from baseline: (A) mean arterial pressure (MAP), (B) heart rate, (C) pulse pressure, (D) cardiac index (CI), (E) stroke volume index (SVI), and (F) total peripheral resistance (TPR).

There were five hemorrhages (3/5 during fast rate, 4/5 during second hemorrhage) resulting in the stopping criteria being reached with less than 25% of blood volume removed. These experiments reached the hemorrhage stopping point with 17.6 ± 1.8% EBVR compared with 33.7 ± 1.9% for the remaining bleeds. MAP responses were similar with respect to EBVR/EBVR_MAX_ for the low‐tolerant hemorrhages (Fig. 7A). Contrary to high‐tolerant animals, the cardiac response was limited (Fig. 7B) and total peripheral resistance decreased (Fig. 7F) during low‐tolerant hemorrhages. This resulted in a pulse pressure that increased during the last 20% of blood volume removed compared to a decrease in pulse pressure as seen in the high‐tolerant hemorrhages (Fig. 7C). The result of these differences was a significant MAP decrease at less volume of blood removal and less change in cardiac index (Fig. 7D) and stroke volume index (Fig. 7F).

## Discussion

The conscious sheep model used in this study was designed to determine if hemorrhage rate affected the early physiologic response leading to hemorrhagic hypotension. Although many large animal hemorrhage studies have been performed to focus on the shock phases of hemorrhage, our objective was to specifically focus on the physiology leading to hypotension and how injury variability, modeled here by a simple change in hemorrhage, is a factor in this response. Seven of the 16 hemorrhages required >30% of blood removal before a significant fall in MAP, highlighting the limitation of using fixed volume hemorrhages when the objective is to monitor this early response. The faster hemorrhage rate induced a stronger tachycardia response at earlier levels of EBVR/EBVR_MAX_ with a lower SvO_2_ and increased lactate, but these differences did not coincide with reduced tolerance as defined in this study. In addition, our primary finding is that on average tolerance to blood loss, defined here as the blood volume loss necessary to decrease MAP by 30 mmHg, was not affected by the hemorrhage rate.

A confounding observation was that the physiologic response during these hemorrhages demonstrated substantial variability with the volume of blood required to induce a 30 mmHg drop in MAP, ranging from 13.3% to 47.8% of total estimated blood volume. Physiological measurements varied considerably between experiments across this spectrum of tolerance, highlighting the challenge to interpreting vital sign measurements during bleeding and “who needs care first” triage scenarios (Schafer et al. 2013). In our study, even knowing the volume of blood that had been withdrawn for each experiment would have been a poor predictor of when a sudden drop in blood pressure would occur. This supports the need for continuous biomarkers that monitor an individual patient's response from, for example, novel sensors, physiological waveform patterns, or by combining information from multiple vital signs to identify compensating patients and provide appropriate therapies (Shackelford et al. 2014). Rahbar et al. identified the potential importance of not only considering the type of transfusion, but also the time of delivering that transfusion (Rahbar et al. 2015). Monitoring and considering the individual physiology could improve this connection.

### Effect of hemorrhage rate on physiologic response

During the fast hemorrhage, preservation of MAP resulted mainly by an increase in heart rate that maintained cardiac output during decreasing stroke volume. Heart rate increased to a lesser degree during the slow hemorrhage which resulted in decreased cardiac output during the later stages of hemorrhage. These differences between the fast and slow occurred with equivalent and consistent stroke volume decreases during hemorrhage. On average, TPR was not altered during the hemorrhage, but when low‐tolerant hemorrhages were excluded there was a rise in TPR toward the end of hemorrhage. Starc et al. observed a heart rate increase early in a fast but not slow hemorrhage in conscious sheep (Starc and Stalcup 1986). This differs from studies in conscious rats that observed a higher heart rate during the compensatory phase for a slower (0.5 mL/kg_BW_/min) compared to faster (2 mL/kg_BW_/min) and intermediate (1 mL/kg_BW_/min) hemorrhages (Ahlgren et al. 2007; Porter et al. 2009). Conscious sheep have stronger cardiac and less vasoconstrictive responses during the compensatory phase of hemorrhage compared to conscious rats and rabbits (Frithiof and Rundgren 2006). That appeared to be the case during our study, but after the initial compensation phase ended, the cardiac response was depressed and an increase in TPR contributed to raising and stabilizing blood pressure. In conscious sheep, the cardiac baroreflex response is the primary driver during compensation while changes in TPR are initiated in the late stages of compensation and help stabilize blood pressure during the decompensatory phase.

An increase in hemoglobin and hematocrit, likely due to splenic contraction, was observed in the 50–90% EBVR/EBVR_MAX_ measurement zone only for the fast hemorrhage. Differences between rates may be due to the timing of intermittent measurements. Contraction may be a relatively rapid response that was not captured at the longer time intervals between measurements during the slow hemorrhage. Posthemorrhage, hemoglobin decreased for both rates indicating dilution due to redistribution of fluids from tissues to vessels (Vaid et al. 2006). This has been observed in human blood loss studies but not lower body negative pressure models of decompensation (Johnson et al. 2014). The redistribution of fluids resulted in increased stroke volume to support cardiac output as heart rate came down and along an increase in TPR that helped to raise and stabilize MAP after both hemorrhage rates.

#### Severity of response

The physiologic response was more severe after the fast hemorrhage as noted by a lower SvO_2_, higher O_2_ER, and increase in arterial lactate production. CVP and pulse pressure were also lower after the fast hemorrhage. This could be due to the experimental challenge of turning off the rotary pump and ending the hemorrhage precisely at the time of a 30 mmHg MAP decrease during the fast hemorrhage. This resulted in a slightly lower minimum MAP after the fast hemorrhage. The longer blood withdrawal period during the slow hemorrhage may also have enabled more vasoconstriction and redistribution from fluids to tissues during the hemorrhage (Bassin et al. 1971), providing a faster recovery of MAP after the slow hemorrhage.

### Tissue oxygenation

The end of the compensatory phase was characterized by a sudden drop in SvO_2_. During human lower body negative pressure experiments, cardiovascular collapse occurs at an O_2_ER of ~55% (Ward et al. 2010). This value was reached at the end the hemorrhages (for slow and fast as well as low‐ and high‐tolerance groups) as SvO_2_ decreased to <45% with the end of compensation. rSO_2‐THIGH1_ and rSO_2‐THIGH2_ were only recorded during a limited number of hemorrhages, but they generally showed a steady decrease in tissue oximetry during both rates. rSO_2‐CEREBRAL_ was relatively stable through most of the hemorrhage, but decreased toward the end of hemorrhage, and continued to decrease posthemorrhage rather than recovering with MAP. This agrees with previous results in anesthetized swine that show cerebral oximetry responses matched MAP responses during hemorrhage (Navarro et al. 2012). Redistribution of blood flow during hemorrhage may limit oxygen delivery to peripheral regions while cerebral autoregulation maintains a sufficient supply of oxygen.

### Hemorrhage model

Our hemorrhage model was designed to investigate the early prehypotensive compensatory response and volume removal necessary to induce a noticeable drop in blood pressure. Although small volume (up to 20%) blood withdrawal studies are often performed in humans, this low level of blood loss does not necessarily result in noticeable physiological changes due to compensation mechanisms (Price et al. 1966). For this reason we performed hemorrhages until a significant drop in MAP from baseline occurred rather than hemorrhaging a fixed volume. We selected a decrease in 30 mmHg based on previous conscious sheep studies and observations that when the compensatory phase ends and there is a considerable MAP decrease, it generally continues to rapidly decrease (Hjelmqvist et al. 1991; Frithiof et al. 2006; Scully et al. 2015). Frithiof et al. have used a similar protocol to examine the effect of opioid receptors on the length of the compensation phase in sheep, bleeding until a MAP of <50 mmHg (Frithiof et al. 2006). Because of the sudden drop in MAP upon the end of the compensatory phase, the minimum MAP we observed came close to this point immediately after the hemorrhage was stopped (56.1 ± 2.8 mmHg across all hemorrhages). However, for two hemorrhages where the baseline MAP was greater than 100 mmHg the minimum MAP reached was 70.4 and 82.6 mmHg. These animals may not have reached the end of the initial compensation phase. For these two animals the percent estimated blood volume removed to reach the stopping point was >30%, so even if a fixed volume hemorrhage was used they would not have reached decompensation.

It should be noted that sheep, being ruminants and genetically distinct from humans, may have different physiologic responses to hemorrhage than humans (Hauser 2005). In terms of hemodynamics, humans have higher hemoglobin and relatively smaller spleens than sheep; so that splenic contraction provides less compensation during hemorrhage than it may in sheep. As observed in our study and others (Frithiof and Rundgren 2006), the heart rate response dominates the compensatory period in sheep while humans may rely more on a combined rising heart rate and vasoconstriction (Cooke et al. 2009; Johnson et al. 2014).

### Individual tolerance

Low‐ and high‐tolerant experiments showed different early compensatory response patterns, with low‐tolerant animals seemingly never entering into a sympathoexcitatory phase. Our study was not designed to directly investigate hemorrhage tolerance, but the observed differences between the low‐ and high‐tolerant groups at the end of the hemorrhage are substantial with divergent TPR and heart rate responses. Low‐ and high‐tolerant human subjects can be distinguished by lower body negative pressure experiments (Rickards et al. 2011). Schafer et al. point to how the lack of a substantial early cardiac response to blood loss challenges the prehypotensive identification of these subjects with standard vital sign‐based indices such as the Shock Index (Schafer et al. 2013). This was true for our low‐tolerant hemorrhages, as there was minimal change in heart rate and pulse pressure increased, rather than decreased, at the end of hemorrhage.

To reduce the total number of animals used in this study, we performed repeated hemorrhages on the same animals. At least 3 days passed between the two hemorrhages, and from our previous experience we did not expect effects from the first hemorrhage day to the second (Rafie et al. 2004; Scully et al. 2015). Hemorrhage tolerance was not significantly different between the days; however, four of the five low‐tolerant hemorrhages occurred during that animal's second hemorrhage. Only a single animal had a low‐tolerant response during both hemorrhages. This suggests there may have been an effect from the first hemorrhage resulting in the potential for less robust compensation mechanisms and lower tolerance to blood loss on the second day of hemorrhage. Future studies using repeated hemorrhage on the same animals should allow more days to pass between hemorrhages.

### Methodological considerations

The use of conscious sheep that were able to move around in their cage resulted in periods of significant noise and at times physical damage to sensors, limiting some of the data available for analysis. Tissue oximetry sensors did not provide usable data for a number of experiments. The pulse‐oximeter sensor attached to the tails of the animals was also malfunctioning (sensitive to movement) during the study resulting in unavailable data for three hemorrhages. However, in the experiments with functional pulse oximetry, SpO_2_ was stable throughout the hemorrhage and posthemorrhage period. This was also observed in the intermittent SaO_2_ blood analyses, including for the animals lacking SpO_2_. There is no reason to suspect that SpO_2_ was not stable for the experiments with missing SpO_2_ data.

Urine output did not significantly change during the hemorrhage; however, there were intermittent sudden increases measured throughout the study likely due to movement of the animal and catheter that allowed fluid build‐up at certain points in the tubing to release. It would be expected that glomerular filtration rate would steadily decrease during the hemorrhage, and there is no reason to expect that the measured increases are physiological.

Besides the hemorrhage induced by the pump, there was additional blood loss resulting from the blood draws (~2 mL for arterial blood gas, ~2 mL for venous blood gas, and ~6 mL for possible future assays). Combined this totaled an additional 10 mL of blood drawn at the intermittent measurements points, but this was offset by thermodilution injections of iced saline (2 × 10 mL). There was a net gain of ~10 mL at each measurement point resulting in an addition of 20–30 mL during the hemorrhage, less than 10% of the volume of blood withdrawn for the experiment with the lowest tolerance.

## Conclusion

We investigated the simple effect of varying the hemorrhage rate on the physiologic response. Our hemorrhage model allowed us to assess the compensatory response leading up to a 30 mmHg decrease in MAP. Although the fast hemorrhage resulted in a stronger cardiac response during the hemorrhage, the hemorrhage rate did not result in substantial differences in tolerance to hemorrhage. Individual tolerance, more so than hemorrhage rate, affected the physiologic response leading up to a drop in MAP. Physiologic responses during low‐tolerant hemorrhages in this study had the most limited changes in vital signs, potentially making them the most difficult to detect. Early vital sign changes are poor monitors and predictors of the response to hemorrhage.

## Conflict of Interest

There are no conflicts of interest. The mention of commercial products, their sources, or their use in connection with material reported herein is not to be construed as either an actual or implied endorsement of such products by the Department of Health and Human Services.
